# Comparison of placenta consumers’ and non-consumers’ postpartum depression screening results using EPDS in US community birth settings (*n*=6038): a propensity score analysis

**DOI:** 10.1186/s12884-023-05852-7

**Published:** 2023-07-22

**Authors:** Daniel C. Benyshek, Marit L. Bovbjerg, Melissa Cheyney

**Affiliations:** 1 Department of Anthropology, Nutrition and Reproduction Laboratory, University of Nevada, Las Vegas, NV USA; 2 Kirk Kerkorian School of Medicine at University of Nevada, NV Las Vegas, USA; 3 Epidemiology Program, College of Public Health and Human Sciences, Oregon State University, Corvallis, OR USA; 4 Department of Anthropology, Oregon State University, Corvallis, OR USA

**Keywords:** Postpartum depression, Placentophagy, Placentophagia, Propensity score analysis, Community birth

## Abstract

**Background:**

Preventing postpartum depression (PPD) is the most common self-reported motivation for human maternal placentophagy, yet very little systematic research has assessed mental health following placenta consumption. Our aim was to compare PPD screening scores of placenta consumers and non-consumers in a community birth setting, using propensity score matching to address anticipated extensive confounding.

**Methods:**

We used a medical records-based data set (*n* = 6038) containing pregnancy, birth, and postpartum information for US women who planned and completed community births. We first compared PPD screening scores as measured by the Edinburgh Postpartum Depression Scale (EPDS) of individuals who consumed their placenta to those who did not, with regard to demographics, pregnancy characteristics, and history of mental health challenges. Matching placentophagic (*n* = 1876) and non-placentophagic (*n* = 1876) groups were then created using propensity scores. The propensity score model included more than 90 variables describing medical and obstetric history, demographics, pregnancy characteristics, and intrapartum and postpartum complications, thus addressing confounding by all of these variables. We then used logistic regression to compare placentophagic to non-placentophagic groups based on commonly-cited EPDS cutoff values (≥ 11; ≥ 13) for likely PPD.

**Results:**

In the unmatched and unadjusted analysis, placentophagy was associated with an increased risk of PPD. In the matched sample, 9.9% of women who ate their placentas reported EPDS ≥ 11, compared to 8.4% of women who did not (5.5% and 4.8%, respectively, EPDS ≥ 13 or greater). After controlling for over 90 variables (including prior mental health challenges) in the matched and adjusted analysis, placentophagy was associated with an increased risk of PPD between 15 and 20%, depending on the published EPDS cutoff point used. Numerous sensitivity analyses did not alter this general finding.

**Conclusions:**

Placentophagic individuals in our study scored higher on an EPDS screening than carefully matched non-placentophagic controls. Why placentophagic women score higher on the EPDS remains unclear, but we suspect reverse causality plays an important role. Future research could assess psychosocial factors that may motivate some individuals to engage in placentophagy, and that may also indicate greater risk of PPD.

**Supplementary Information:**

The online version contains supplementary material available at 10.1186/s12884-023-05852-7.

## Introduction

Postpartum depression (PPD) is a significant, and potentially severe, health problem [1] in the US and around the world. A recent systematic review (565 studies from 80 countries/regions) estimated the prevalence of PPD in the US of 18.5%, with an average global prevalence of 17.2% [2]. The high prevalence is made more acute by cumulative shortcomings in clinical recognition, initiation and adequacy of treatment, as well as in treatment response and the lack Food and Drug Administration (FDA)-approved treatment options for PPD [3]; collectively these are known as the “perinatal depression treatment cascade.” [4] The current, sole FDA-approved treatment is expensive, administered as an intravenous infusion, and requires multiday hospitalization. In addition, concerns over negative side effects and risks of commonly prescribed, off-label psychotropic medications in the postpartum period [5, 6], especially among women who breastfeed their infants [3], are additional challenges to effective treatment. Although a variety of interventions aimed at preventing PPD have been proposed, rigorous studies evaluating their effectiveness are limited, especially across a range of settings and participant populations.

In 2019, the US Preventive Services Task Force (USPSTF) made new recommations on interventions to prevent depression during pregnancy and following childbirth [7]. The task force reviewed a wide range of interventions, including those based on counseling, physical exercise, education programs, social support, and behavioral interventions, among others, in addition to pharmacological approaches. The task force concluded that counseling interventions, including cognitive behavioral and interpersonal therapies, are effective in preventing perinatal depression. It also concluded that pregnant or postpartum women at increased risk for perinatal depression due to a history of depression, current depressive symptoms, and other factors, are likely to benefit from counseling interventions. The task force found insufficient evidence to assess the benefits and harms of noncounseling interventions, and recommended that “clinicians provide or refer pregnant or postpartum persons who are at increased risk for perinatal depression to counseling interventions.” While the evidence of the effectiveness of counseling interventions were characterized as “convincing,”with a 39% (pooled) reduction in the likelihood of experiencing perinatal depression across studies, the recommendations have been critiqued as requiring access to mental health resources out of reach for many pregnant and postpartum people [8, 9]. In addition, although the USPSTF review included a small number of psychosocial interventions that may also be effective, but which are less time intensive and more easily and affordably accessed, these studies, like those evaluating the effectiveness of prophylactic treatment with antidepressant medications for the prevention of PPD, were of insufficient quantity and/or quality to be recommended by the task force [7].

Because of the lack of availability of mental health counseling services, combined with few pharmaceutical options, some individuals with concerns about postpartum mood disturbances turn to alternative/complementary health-seeking practices. Maternal placentophagy is one such alternative/complementary practice in the US and other high-resource countries. Individuals who engage in the practice consume their placentas, typically in the first days or weeks after birth, for a host of purported maternal health benefits that are believed to be derived from the organ’s nutritional and biochemical properties [10]. Multiple self-report surveys in the US [10], UK [11], and Canada [12], identify improved postpartum mood, including the prevention of PPD, as the overwhelming primary motivation for engaging in maternal placentophagy. Benyshek and colleagues [13] report that among 7162 placentophagic individuals in US community birth settings (i.e., at home or a birthing center) for whom the reasons for placentophagy was known to the midwife, 4331 (73%) reported improved postpartum mood as the primary motivating factor. That study, which is the largest to date of placentophagic mothers (*n*= 7162), revealed the following demographic profile: a mean age of 31 years; predominantly non-Hispanic white ethnicity (84%), college educated with at least a bachelor’s degree (55%), in a married/partnered relationship (94.8%), and with no history of clinically treated pregravid anxiety or depression (92%) [13]. While maternal placentophagy is most closely associated with the home birth movement in the US [13–15], the practice is not limited to those who give birth in non-clinical settings. A 2013 survey (*n*= 189) showed that while a majority (66%) of the placentophagic respondents gave birth at home (or birthing center), fully one-third (34%) gave birth in a hospital [10].

While a small number of rigorous scientific studies and case reports have assessed the potential health risks [13, 16–18] and benefits of maternal placentophagy, there is currently no clear clinical evidence of maternal or neonatal harm [13, 19] or benefit [19–21]. The present study extends the limited research on human placentophagy by employing propensity score (PS) analysis utilizing the Midwives Alliance of North America (MANA) data set to investigate the association between maternal consumption of the placenta and improved postpartum maternal mood — the most commonly cited self-reported benefit of the practice.

Beyond a handful of qualitative, self-report studies of placentophagic individuals [10–12], and a single, phase 1 randomized clinical trial (RCT) which assesed the safety and therapeutic efficacy of maternal placentophagy on a number of maternal outcomes (*n*= 27) [19–21], the only rigorous scientific study that has investigated the potential effect of human placenta consumption on maternal postpartum mood is a 2018 matched retrospective cohort study drawn from a sample of birthing people with a history of mood disorders [22]. Like the phase 1 RCT, it included a small sample of placenta consumers (*n* = 28) and is limited by very low statistical power and a concurrent inability to adequately control for confounders. Given the established and growing alternative maternal health practice of maternal placentophagy, additional research that can better assess the safety and efficacy of the practice are currently needed.

In the present study, our hypothesis was that, in unadjusted analyses, women who consumed their placentas would report higher PPD screening scores, as measured by the Edinburgh Postpartum Depression Scale (EPDS), than those who did not, given that previous research has shown that placenta consumers were more likely to report pregravid anxiety or depression than placenta non-consumers [13]. However, in adjusted analyses, we anticipated there would be no difference in PPD screening scores between placenta consumers and non-consumers.

## Methods

### Population and sample

Data come from the MANA Stats dataset, birth years 2016–2018; reliability and validity of the data are presented elsewhere [23]. This dataset contains complete course of care information for birthing people who planned community births with midwives who participate in the MANA Stats data collection project. MANA Stats captures approximately 20% of planned home births in the US, and 15% of planned birth center births [24]. Within practices of contributing midwives, more than 98% of women agree to have their de-identified data entered into MANA Stats [25]. All study procedures were approved by the Oregon State University Institutional Review Board. Both midwives and clients provided informed consent for their MANA Stats data to be used in research.

The identified birth years were chosen because questions on postpartum depression were added in 2016. From the entire sample, we limited the dataset to those women who planned and completed a community birth (i.e., we dropped individuals who began labor intending a home or birth center birth but then transferred to a hospital during labor), because women birthing in the hospital are less likely to be able to obtain their placentas – for consumption or other reasons. We further excluded twin pregnancies, those affected by intrapartum or neonatal death, and those missing data on either the placentophagy (exposure) variable or the postpartum depression (outcome) variable. The final sample size for the main analysis, after applying these criteria, was *n* = 5974.

### Exposure: placentophagy

Midwives were asked whether the client consumed their placenta. Answer options were “yes,” “no,” or “I don’t know.” For the main analysis, we limited the dataset to those people for whom a definitive yes or no answer was provided by the midwife. Additional details were collected about preparation methods for those individuals who did consume their placentas; these results are reported elsewhere [13].

### Outcome: postpartum depression

Beginning in 2016, midwife contributors to MANA Stats were asked whether or not they had used the EPDS to screen for mental health challenges during the postpartum period. If a midwife answered “yes,” she was asked to report that client’s score. Midwives who used the EPDS tool did so at the last postpartum visit, which typically occurs at 6–8 weeks postpartum [26]. To define PPD using EPDS, we used two cutoff points advocated in the literature: ≥ 11 and ≥ 13 [27, 28]. All analyses were conducted twice, once using each of these cut points; we report results from both throughout.

### Data analysis

Propensity score (PS) matched analysis allows researchers to mimic an experimental study design using data from an observational study when an experimental study design (e.g., RCT) is infeasible or unethical. Compared to RCT experimental studies, which aim to ensure participant characteristics are completely comparable across treatment groups via randomization, observational studies must achieve this aim adjusting the analysis for differences between comparison groups. PS analysis controls for differences in participant characteristics between study groups by carefully matching the measured covariates between exposure groups, allowing for more confounder control than available with traditional cohort analyses [29, 30].

In the present study, we first compared (unmatched) individuals who consumed their placenta to those who did not on demographics, pregnancy characteristics, history of mental health challenges, and PPD. These unadjusted analyses used chi-square and t-tests. We then created matching exposed/unexposed groups using propensity score methods. The propensity score model included more than 90 variables describing medical and obstetric history, demographics, pregnancy characteristics, and intrapartum and postpartum complications; see Table 1. To avoid listwise deletion of records when running models with that many covariables, missing data were either assigned to their own category or imputed; see Additional file 1: Appendix 1 for details. The matching protocol included frequency matching within decile of propensity score. The matching algorithm and subsequent analyses were repeated 1000 times with standard errors estimated using standard bootstrap methods.

**Table 1:** Variables included in the propensity score generation model

category	included variables
maternal demographics	race/ethnicity, partner status, education, expected payment source, WIC eligibility, maternal age, pre-gravid BMI, region of residence [New England, mid-Atlantic, Southeast, Midwest, Southwest, West], whether the mother was Amish
obstetric history	gravidity, parity, history of cesarean, history of vaginal birth, history of other cervical surgery, history of other uterine surgery
pre-gravid history of chronic diseases	asthma, genetic disorders, hypothyroidism, frequent urinary tract infections, sexually-transmitted infections
pre-gravid history of psychosocial complications	anxiety, depression, eating disorders, domestic violence, sexual abuse, substance abuse
prenatal care characteristics	credentials held by the attending midwife, actual place of birth (home or birth center), number of prenatal visits
pregnancy complications	anemia, asthma, cholestasis, congenital anomalies diagnosed antenatally, spontaneous abortion of a twin with continuing pregnancy, group B strep infection, heart disease, hepatitis B or C infection, hyperemesis, hyperthyroid, hypothyroid, intrauterine growth restriction, urinary tract infection, oligohydramnois, polyhydramnios, preterm labor or premature rupture of membranes, post-term pregnancy, unexpected preterm birth diagnosed by clinical exam of the newborn, placenta previa, placental abruption, puritic urticarcical papules and plaques of pregnancy, Rh sensitivity, sexually-transmitted infections, genital herpes, single umbilical artery
psychosocial complications during pregnancy	anxiety, depression, eating disorder, sexual abuse, substance abuse, domestic violence
intrapartum characteristics and complications	whether an induction was attempted, either pharmacologic or non-pharmacologic, cord prolapse, maternal dehydration, malposition/malpresentation, maternal exhaustion, maternal request for pharmacologic pain relief, maternal shock, light meconium, thick or particulate meconium, non-reassuring fetal heart tones, hypertension, pre-eclampsia, prolonged rupture of membranes with or without labor, urine retention, maternal pushing position, failure to progress in the first stage, failure to progress in the second stage, length of active labor, length of pushing, length of time membranes were ruptured
postpartum and birth outcomes	gestational age at birth, birthweight, hemorrhage > 1000 cc, severe perineal trauma (3^rd^ or 4^th^ degree), any congenital anomalies, transfer to a hospital in the first 6 h for a maternal indication, transfer to a hospital in the first 6 h for a neonatal indication, NICU admission in the first 6 weeks, other neonatal hospitalization in the first 6 weeks, maternal hospitalization in the first 6 weeks

Once the matched samples were generated, we first confirmed that matching had worked as anticipated by comparing exposed to unexposed women on a variety of demographic, pregnancy characteristic, and mental health history variables. We then compared exposed to unexposed for the two screening cutoff values for PPD, using logistic regression models that controlled for the raw propensity scores. This allowed us to account for potential residual confounding secondary to frequency matching. Because the idea that placentophagy can prevent PPD is well known within community birth circles in the US [13], we then re-ran the models additionally controlling for three mental health history variables. These variables had been included in the propensity score generation model (and thus should have been equally distributed between exposed and unexposed groups, thereby eliminating confounding), but because they are potentially very strong confounders, we were concerned about possible residual confounding and thus controlled for them explicitly. In all cases, results from propensity score analyses are presented as the summary from the 1000 bootstrapped matched samples.

We then undertook a series of sensitivity analyses to assess the robustness of our results. We first assumed that all women for whom the midwife did not know whether the placenta had been consumed *did* consume their placentas. Next, we then assumed they all had *not *consumed their placentas. Finally, we limited the exposed group to those women who had consumed the placenta without cooking/steaming (i.e., either unprocessed – “raw” and frozen – or after low temperature-dehydration only), given recent analyses showing extremely large reductions in placental hormonal concentrations when the placenta is cooked (i.e., steamed) prior to low heat dehydration and encapsulation [31]. Under each of these scenarios, we repeated the entire analysis comparing the exposure to the outcome. We re-ran the propensity score model with more women either in the exposed or unexposed group, and then re-ran the bootstrap sampling procedure, and re-calculated the odds ratios and 95% confidence intervals. These analyses were approved by the IRB at Oregon State University. Analyses were conducted using SPSS version 24.0.0 (IBM Corp., Armonk, NY) and R version 3.3.2 (R Foundation for Statistical Computing, Vienna, Austria).

## Results

As expected, women who consumed their placentas differed in many ways from those who did not (see the unmatched columns in Table 2). In the unmatched data, the overall frequency of PPD using the ≥ 11 cut point was 8.2% and 4.6% using the ≥ 13 cut point. As anticipated, in the unmatched and unadjusted analysis, placentophagy was associated with an increased risk of PPD screening score above the cutoff value: OR (95% CI) 1.47 (1.22—1.77) and 1.42 (1.11—1.81) for ≥ 11 and ≥ 13, respectively.

**Table 2:** Comparison of demographic and pregnancy risk factor variables between women who had ate their placenta, compared to women who did not, before and after propensity score matching. The “before” data are presented as percents and frequencies from the dataset as a whole, whereas the “after” data are the percents with 95% confidence intervals, from the propensity score frequency matched datasets that were resampled 1000 times (see methods). Data come from the Midwives Alliance of North America Statistics Project (MANA Stats), birth years 2016–2018

	***Before Matching***			***After Matching***		
Comparison Variable	Ate placenta n (%)	Did not eat placenta n (%)	*p*-value (Chi-square test)	Ate placenta % (95% CI)	Did not eat placenta % (95% CI)	*p*-value (Chi-square test)
**Total**	***n* = 2065**	***n* = 3973**		***n* = 1876**	***n* = 1876**	
Midwife-identified race of mother = White	1711 (83.7%)	3370 (85.8%)	0.017	84.4% (84.0 – 84.9)	84.5% (83.7 – 85.4)	0.93
Mother is married or partnered	1939 (94.8%)	3763 (95.8%)	0.049	94.9% (94.7 – 95.2)	95.1% (94.6 – 95.6)	0.76
Maternal education BA/BS or more	1109 (54.8%)	1815 (46.5%)	< 0.001	53.6% (53.1 – 54.1)	54.0% (52.9 – 55.1)	0.82
Mother eligible for WIC during this pregnancy	203 (9.9%)	389 (9.9%)	1.0	10.0% (9.6 – 10.2)	9.9% (9.2 – 10.7)	0.91
Mother eligible for Medicaid	456 (22.3%)	952 (24.2%)	0.048	22.7% (22.3 – 23.1)	23.1% (22.1 – 24.0)	0.79
Place of birth						
home	1375 (67.2%)	2106 (53.6%)	< 0.001	65.2% (64.9 – 65.6)	65.7% (64.4 – 67.0)	0.73
birth center	671 (32.8%)	1822 (46.4%)		34.8% (34.4 – 35.1)	34.3% (33.0 – 35.6)	
Primary midwife credential^a^						
Certified Professional Midwife	1267 (61.9%)	2254 (57.4%)	< 0.001	61.0% (60.1 – 61.5)	61.1% (59.9 – 62.4)	0.96
Certified Nurse Midwife	349 (17.1%)	844 (21.5%)		17.9% (17.5 – 18.1)	17.6% (16.6 – 18.7)	
Other	430 (21.0%)	830 (21.1%)		21.2% (20.7 – 21.5)	21.2% (20.1 – 22.3)	
Pre-gravid BMI						
< 18.5	74 (3.6%)	138 (3.5%)	0.049	3.7% (3.5 – 3.8)	4.0% (3.6 – 4.5)	0.99
18.5—< 25	1286 (63.4%)	2339 (59.8%)		62.4% (61.9 – 62.9)	62.0% (60.8 – 63.3)	
25—< 30	418 (20.6%)	889 (22.7%)		20.7% (20.3 – 21.1)	20.8% (19.8 – 21.9)	
30—< 35	161 (7.9%)	327 (8.4%)		8.1% (7.8 – 8.3)	8.1% (7.4 – 8.8)	
35 +	90 (4.4%)	220 (5.6%)		4.5% (4.3 – 4.6)	4.5% (4.3 – 4.6)	
Mother is primiparous	645 (31.5%)	1008 (25.7%)	< 0.001	30.7% (30.1 – 31.2)	30.2% (29.0 – 31.2)	0.75
If multipara, history of cesarean^b^						
and history of vag. birth	52 (3.7%^c^)	110 (3.8%^c^)	0.068	3.7%^c^ (3.5 – 4.0)	3.8%^c^ (3.2 – 4.4)	0.99
without history of vag. birth	42 (3.0%^c^)	55 (1.9%^c^)		2.8%^c^ (2.6 – 3.1)	2.8% (2.3 – 3.2)	
Mother has “significant psychosocial history”^d^	695 (34.0%)	940 (23.9%)	< 0.001	32.4% (31.9 – 32.9)	31.2% (30.1 – 32.2)	0.42
Mother has history of anxiety, depression of psychiatric disease, treated with drugs or inpatient therapy	218 (10.7%)	303 (7.7%)	< 0.001	14.0% (13.2 – 14.7)	13.9% (12.7 – 15.1)	0.92
Mother has history of anxiety, depression of psychiatric disease, NOT treated with drugs or inpatient therapy	419 (20.6%)	575 (14.7%)	< 0.001	23.3% (22.5 – 24.0)	23.3% (22.1 – 24.6)	1.0
	**mean (SD)**	**mean (SD)**		**mean (95% CI)**	**mean (95% CI)**	
Maternal age	31.0 (4.8)	30.3 (4.9)	< 0.001^e^	30.9 (30.9 – 31.0)	30.9 (30.8 – 31.0)	1.0
Gestational age, days^f^	281.4 (7.4)	280.8 (7.7)	0.006^e^	281.3 (281.2 – 281.3)	281.3 (281.1 – 281.5)	1.0

^a^Certified Nurse Midwife category includes one birth attended by a midwife holding both the CPM and CNM credential. “Other” includes students attending as primary but under supervision, clinicians with other credentials (e.g., Naturopathic Doctor, Doctor of Osteopathy), and records missing midwife credential information

^b^all women in this group had a vaginal birth because the sample was limited to completed community births

^c^denominator is multiparas only

^d^presence of any of the following in the medical history: anxiety/depression/psychiatric disease, treated with drugs or inpatient therapy; anxiety/depression/psychiatric disease, not treated with drugs or inpatient therapy; eating disorders; domestic violence; sexual assault, substance abuse

^e^for maternal age and gestational age, the *p*-values are from a t-test assuming equal variances

^f^preterm birth is contraindicated in the community setting; thus the mean gestational age is longer than would be expected in the population as a whole

The propensity score matching protocol worked as expected, and in the matched dataset no differences were observed between exposed and unexposed women (see matched columns in Table 2). However, contrary to our (null) hypothesis, controlling for these 91 variables via propensity score matching did not completely attenuate the association. Indeed, regardless of whether the models additionally controlled explicitly for history of mental health challenges, placentophagy was associated with an increased risk of PPD of 20% if the ≥ 11 cut point was used, and 15% if ≥ 13 was used (see main analysis rows in Table 3). None of the sensitivity analyses altered this general finding (see Table 3).

**Table 3:** Adjusted comparisons for 2 definitions of postpartum depression as measured by the Edinburgh Postnatal Depression Scale (EPDS), comparing women who ate their placenta to women who did not, using bootstrapped propensity-score matched data from MANA Stats, birth years 2016–2018. Absolute risks (proportions), adjusted odds ratios, and the corresponding 95% confidence intervals were calculated from the bootstrapped samples

Outcome	Ate Placenta % (95% CI)	Did not eat placenta % (95% CI)	Model 1^a^ aOR (95% CI)	Model 2^b^ aOR (95% CI)
**Main analysis**	***n* = 1876**	***n* = 1876**		
EPDS ≥ 11	9.9% (9.6 – 10.2)	8.4% (7.8 – 9.0)	1.20 (1.10 – 1.31)	1.19 (1.09 – 1.30)
EPDS ≥ 13	5.5% (5.3 – 5.8)	4.8% (4.4 – 5.3)	1.15 (1.03 – 1.29)	1.14 (1.02 – 1.28)
**Assuming unknown is yes**	***n* = 2532**	***n* = 2532**		
EPDS ≥ 11	9.2% (8.8 – 9.6)	7.8% (7.3 – 8.3)	1.19 (1.09 – 1.29)	1.18 (1.09 – 1.28)
EPDS ≥ 13	5.3% (4.9 – 5.6)	4.5% (4.1 – 4.8)	1.19 (1.06 – 1.33)	1.17 (1.05 – 1.31)
**Assuming unknown is no**	***n* = 1932**	***n* = 1932**		
EPDS ≥ 11	10.0% (9.7 – 10.3)	8.9% (8.1 – 9.6)	1.13 (1.03 – 1.25)	1.13 (1.03 – 1.25)
EPDS ≥ 13	5.6% (5.3 – 5.8)	5.1% (4.5 – 5.6)	1.10 (0.98 – 1.25)	1.10 (0.97 – 1.25)
**Only counting raw**	***n* = 1080**	***n* = 1080**		
EPDS ≥ 11	10.8% (10.5 – 11.2)	8.3% (7.2 – 9.4)	1.33 (1.17 – 1.56)	1.34 (1.16 – 1.57)
EPDS ≥ 13	6.3% (5.9 – 6.5)	4.8% (4.0 – 5.7)	1.31 (1.08 – 1.61)	1.30 (1.07 – 1.61)

^a^Model 1 used propensity score-matched data, and controlled additionally for the actual propensity scores, to adjust for any residual confounding subsequent to the frequency matching procedure

^b^Model 2 controlled additionally for “significant” psychosocial history (see footnote to Table 1), history of anxiety or depression treated with drugs or inpatient therapy, and history of anxiety or depression not treated with drugs or inpatient therapy. These variables were all included in the propensity score model, and based on Table 1, indeed ended up equally matched between the two groups, but always the possibility of residual confounding, especially for such important covariables

## Discussion

Multiple studies have established that the primary motivation, among individuals that have engaged in placentophagy, was improved postpartum maternal mood and prevention of PPD [10–13]. The sole phase 1 RCT (*n*= 27) investigating the safety and potential therapeutic efficacy of placentophagy on maternal mood (and other maternal outcomes) found no significant main effects related to maternal mood between participants consuming steamed and dehydrated placenta versus a placebo; however, small, time-related improvements in maternal mood post-supplementation (in the first week postpartum) among placenta group participants were observed, though this study was underpowered [20]. Similarly, in a 2018 matched retrospective cohort study drawn from a sample of women with a history of mood disorders, Morris and colleagues [22] reported no significant differences in postpartum mood (as measured by EPDS or Sleep–Wake Activity Inventory) between non-placenta consuming control participants (*n* = 110) and placenta consumers (*n* = 28). Again, however, small numbers suggest very low statistical power. The present study extends these preliminary findings, and benefits from both its robust sample size and the large number of variables matched between the two exposure groups in the PS analysis. The main finding reported here, which shows a higher PPD screening score among matched placenta consumers versus non-consumers in the adjusted analysis, remains to be explained.

By carefully matching the placenta consuming and non-consuming cohorts on a wide array of variables, many potential confounds in the unadjusted data were mitigated, including the larger percentage of placenta consumers who report pregravid anxiety or depression (both ‘treated’ and ‘not treated’ with medication or inpatient therapy). Given that higher rates of PPD among those who consumed their placenta persisted nonetheless, it is possible that our findings indicate confouding by indication or reverse causality — that is, those who were most depressed, or at highest risk for depression, were more likely to consume their placenta. History of mood disorders, and therefore risk for PPD, may have been poorly measured in MANA Stats. Specifically, while our analysis matched on pregravid mental health risk factors, the questions were not sufficiently nuanced to capture severity of pre-existing conditions, nor other key predictors of postpartum mental health such as perceived social support [32, 33], spousal support [34], stress [35, 36], sleep duration/quality [37], or breastfeeding difficulties [38].

In addition, social stigma associated with mental health challenges [39] (especially while parenting), may further complicate accurate assessment of preexisting depression and anxiety rates (let alone severity). It is likely, for example, that some individuals answered “no” to pregravid mental health issues due to stigma or to underdiagnosis as a result of widespread barriers to accessing mental health services. The EPDS was also conducted at 6–8 weeks postpartum in most instances, and thus true rates of PPD (with onset occuring anytime in the first year postpartum) may be underestimated given the relatively early administration of the EPDS [2]. Significant differences between the two cohorts (during a current or previous postpartum period) for any or all of these variables could help explain both an individual’s decision to consume the placenta in the hopes of preventing postpartum mood disturbances (or another purported benefit), *and* the higher average EPDS score among consumers, as all of these factors have been linked to increased risk of postpartum mood disturbances. Ironically, inadequate mental health services in the US may contribute to both inaccurate/under reporting *as well as* the desire to consume one’s placenta.

Another, related, issue may be historical and ongoing treatments for mood disorders. If people in our sample who ate their placentas did so in an attempt to reduce their risk of PPD, it is possible this is a group of people who would decline more traditional pharmacologic management of mood disorders in favor of so-called “natural” remedies (like placentophagy). We might, therefore, have a sample in whom baseline mood disorders are less well controlled than they might otherwise have been.

Lastly, it is also possible that consumption of the placenta does somehow contribute to higher rates of PPD — that is, placentophagy could *cause* or *exacerbate* postpartum mood disturbances. While this currently cannot be ruled out, we suggest that a combination of confounding by indication/reverse causality, social stigma, inadequate mental health services, and/or limits of the data collection tool are more likely explanations for our results. Regardless, our findings clearly suggest that psychosocial factors are of critical importance in helping to explain why some individuals choose to eat their placenta. Beyond additional future RCT studies that could assess potential casual effects of maternal placentophagy on postpartum mood, additional studies are needed to identify the social and psychological factors that lead some individuals to engage in placentophagy. We also need better information on PPD mitigation strategies, the absence of which may also help explain greater risk for PPD among placenta consumers.

### Strengths and limitations

The main strengths of our study are the large sample size (orders of magnitude larger than previous efforts) and the robust propensity score matching that allowed for control of more than 90 potential confounders. However, our study also has several limitations. The data did not include ‘baseline’ prepartum EPDS scores (e.g., 36^th^ week of preganancy) [14]. We were also not able to precisely control for all the potential variation in processing methods of the placenta (e.g., unprocessed/frozen, dehydrated only, steamed and dehydrated, etc.). Thus, while our analyses included limiting the exposed group to those women who had consumed the placenta without cooking/steaming (i.e., “raw”), we were not able to further distinguish between “raw” processed subtypes (e.g., unprocessed/frozen from low temperature dehydrated/pulverized/encapsulated). In addition, neither the amount of placenta consumed per day, the number of times per day, nor the time of day of consumption (i.e., dosage), was reported, and could therefore not be included in our analyses. Variability in preparation and dosing could have affected our findings. As described above, the variables for measuring history of mood disorders were less than ideal. Our pattern of results could be entirely explained by residual confounding and reverse causality. Additionally, we assessed PPD at the 6–8 week, final postpartum visit; it is possible our results would look different if we had EPDS data from 3–6 months postpartum, when we would expect more cases of incident PPD. It is also worth remembering the study represents a sample of community births, which by definition are much lower risk of PPD than a corresponding sample from hospital births (e.g., there are no cesareans in our sample). Findings could have been different with a more representative US sample. However, because the group most likely to engage in placentophagy in the US are people who plan home births, this distinction is likely moot. Finally, we acknowledge that the mental health variables identified in the study may, regrettably, represent some confusing categorical overlap between variables for some readers (e.g., “depression” is actually subsumed under the broader variable of “psychiatric disorder”). Identifying these variables as such was unavoidable, however, as these are the variables as identified in the MANA Stats tool.

### Clinical Implications

Our findings indicate that it is reasonable to question how well we can know a client’s mental health history given widespread social stigma, systems level shortcomings in access to services and under- or mis-diagnosis of mental health conditions, and limitations of existing data collections tools that typically track a very small number of postpartum factors and outcomes. These findings also make clear the importance of screening for factors associated with PPD risk beyond history of mental health challenges, mode of delivery, and breastfeeding, and include social and spousal support, stress, sleep duration/quality, and breastfeeding difficulty. There are important conversations to be had on how to talk about placentophagy with clients, especially those who are highly motivated due to past trauma to “try anything” to avoid PPD.

At this time, there is no evidence that placenta consumption lowers risk of PPD. Providers who might encounter individuals planning to consume their placentas need to address this with their clients. Perhaps most importantly, critical inequities and deficiencies in mental health services in the US need to be addressed. All postpartum people should have access to expanded mental health services that offer a range of effective strategies for preventing and treating postpartum mood disorders.

## Conclusion

While the propensity score matched study design improved our ability to explain the causal relationship between placentophagy and postpartum mood, our findings raise as many questions as they answer. We found that individuals who consumed their placenta scored higher on an EPDS screening than carefully matched non-placentophagic controls. Why placentophagic women score higher on the EPDS despite extensive PS matching remains unclear, but we suspect reverse causality plays an important role. Findings highlight the need to reliably measure factors associated with PPD risk that go beyond history of mental health challenges, mode of delivery, and breastfeeding, as these may be critical for teasing out potentially complex relationships between placentophagy and postpartum mental health.

## Supplementary Information

**Additional file 1. **

## Data Availability

The data that support the findings of this study are available from MANA Stats, but restrictions apply to the availability of these data, which were used under license for the current study, and so are not publicly available. Data are, however, available from author MB (Marit.Bovbjerg@oregonstate.edu) upon reasonable request, and with permission of MANA datasets (research.applications@manastats.org).

## Abbreviations

PPD: Postpartum Depression
EPDS: Edinburgh Postpartum Depression Scale
PS: Propensity Score
MANA: Midwives Alliance of North America
RCT: Randomized Controlled Trial
IRB: Institutional Review Board
