# Supplementary material for: Comparison of placenta consumers’ and non-consumers’ postpartum depression screening results using EPDS in US community birth settings (n=6038): a propensity score analysis
Source: BMC Pregnancy Childbirth. 2023 Jul 22;23:534. doi: 10.1186/s12884-023-05852-7 (PMC10362633; doi:10.1186/s12884-023-05852-7)
Supplement: Supplementary file 1 — Additional file 1. [file 12884_2023_5852_MOESM1_ESM.docx]

**Appendix 1**

For the propensity score generation, missing was a category, unless (i) there were <20 missings (in which case the missings were set to the most frequent category) or (ii) the variable was going to be continuous in the eventual propensity score model. In the latter case, if the variable was quasi-continuous (eg, takes discrete, integer values—eg maternal age), then again missings were set to the most common category. For truly continuous variables, the missings were set to the sample median. BMI was treated as a categorical variable, rather than continuous, because of the high proportion of missingness. Additionally, if there were fewer than 20 events for one of the dichotomous propensity model predictor variables, that variable was combined with a similar one. Details are provided below.

Because this data cleaning was undertaken in order to facilitate propensity score generation, all numbers below are from the unmatched sample (total n=5974).

*Categorical variables with more than 0 but fewer than 20 missings; missing data set to most frequent category*

- Client is married or partnered
  - 1 missing set to “yes”
- Midwife credential
  - one MW who had both a CPM and a CNM credential was moved to the CNM category
- Region
  - one missing set to “Region 6” (west coast)
- Race
  - 10 missing set to “white”
- Mother eligible for WIC during this pregnancy
  - 12 missing set to “no”
- NICU admission in the first 6 weeks
  - 7 missing set to “no”
- Infant hospitalization in the first 6 weeks
  - 6 missing set to “no”
- Maternal hospitalization in the first 6 weeks
  - 3 missing set to “no”
- Severe (3^rd^ or 4^th^ degree) perineal trauma
  - 15 missing set to “no”

*Ordinal variables treated as continuous in the propensity score model; missing data set to most frequent category*

- number of prenatal visits
  - 27 missing set to “12”
- maternal age
  - 2 missing set to “32”

*Variables combined so that there were at least 20 “events” per group*

- History of chronic hypertension (n=10)
- History of pre-existing diabetes (n=2)
- Complications of the index pregnancy: heart disease, symptomatic (n=1)
- Complications of the index pregnancy: hepatitis, chronic B or C (n=3)
- Complications of the index pregnancy: hyperthyroid (n=14)
- Complications of the index pregnancy: anomaly diagnosed during pregnancy (n=11)
- Complications of the index pregnancy: spontaneous abortion of twin, with continuing pregnancy (n=4)
- Complications of the index pregnancy: Intrauterine Growth Restriction (n=3)
- Complications of the index pregnancy: Single umbilical artery (n=15)
- Complications of the index pregnancy: oligohydramnios (n=8)
- Complications of the index pregnancy: cholestasis (n=3)
- Complications of the index pregnancy: Rh sensitization (n=17)
- Complications of the index pregnancy: pruritic urticarial papules and plaques of pregnancy (n=12)
- Complications of the index pregnancy: HIV (n=1)
- Complications of the index pregnancy: STIs other than genital herpes (n=17)
- Complications of the index pregnancy: genital herpes (n=18)
- Complications of the index pregnancy: placental abruption (n=2)
- Intrapartum complications: placental abruption (n=7)
- Intrapartum complications: cord prolapse (n=2)
- Intrapartum complications: pre-eclampsia diagnosed during labor (n=1)
- Intrapartum complications: pregnancy-induced hypertension diagnosed during labor (n=11)
- Intrapartum complications: maternal shock (n=6)
- Intrapartum complications: maternal need for pain relief not available in community setting (n=4)
- Intrapartum complications: urinary retention (n=17)
- History of eating disorders (n=131)
- Complications of the index pregnancy: eating disorders (n=14)
- History of domestic violence (n=121)
- Complications of the index pregnancy: domestic violence (n=18)
- History of sexual abuse or assault (n=250)
- Complications of the index pregnancy: sexual abuse or assault (n=14)
- History of substance abuse (n=67)
- Complications of the index pregnancy: substance abuse (n=13)

*Continuous variables, missing data set to sample median. For labor duration variables, this was done separately for primiparas and multiparas.*

- duration of active labor
  - among primiparas, 15 missing set to the median, 380 minutes
    - this changed the sample mean for primiparas from 482.1 to 481.1
  - among multiparas, 57 missing set to the median, 191 minutes
    - this changed the sample mean for multiparas from 252.4 to 251.6
- duration of ruptured membranes
  - among primiparas, 16 missing set to the median, 182 minutes
    - this changed the sample mean for primiparas from 422.9 to 420.5
  - among multiparas, 45 missing set to the median, 30 minutes
    - this changed the sample mean for multiparas from 208.8 to 207.0
- duration of pushing
  - among primiparas, 17 missing set to the median, 62 minutes
    - this changed the sample mean for primiparas from 85.6 to 85.3
  - among multiparas, 41 missing set to the median, 12 minutes
    - this changed the sample mean for multiparas from 23.2 to 23.1
- Birthweight
  - 4 missing set to sample median of 3600g
    - this did not change the sample mean (3604 g)
- Gestational age (in days)
  - 3 missing set to sample median of 281 days
    - this did not change the sample mean (281.0 days)
